# The Lack of a Glucose Peak During the Oral Glucose Tolerance Test in Pregnancy: What Does It Portend for Perinatal Outcomes?

**DOI:** 10.3390/nu17111785

**Published:** 2025-05-24

**Authors:** Anna Maria Marconi, Nikita Alfieri, Emanuele Garzia, Stefano Terzoni, Stefano Manodoro, Patrick M. Catalano

**Affiliations:** 1 Department of Obstetrics and Gynecology, San Paolo Hospital Medical School, ASST Santi Paolo e Carlo, Via A di Rudinì 8, 20142 Milan, Italy; nikita.alfieri@asst-santipaolocarlo.it (N.A.); emanuele.garzia@asst-santipaolocarlo.it (E.G.); stefano.manodoro@asst-santipaolocarlo.it (S.M.); 2 Department of Health Sciences, University of Milan, 20124 Milan, Italy; 3 Department of Biomedical Sciences for Health, University of Milan, 20124 Milan, Italy; stefano.terzoni@unimi.it; 4 Reproductive Endocrinology Unit, Massachusetts General Hospital, Harvard Medical School, 55 Fruit Street, Boston, MA 02115, USA; pcatalano2@mgh.harvard.edu

**Keywords:** flat oral glucose tolerance test, pregnancy outcome, oral glucose tolerance test, birthweight, gestational diabetes

## Abstract

**Background**: An univocal definition for a lack of glucose peak during the oral glucose tolerance test in pregnancy (flat curve) has never been agreed upon. Thus, the aim of this study was to provide a definition for the flat 75 g oral glucose tolerance test (OGTT) and to assess its clinical significance. **Methods**: A retrospective cohort study, where 8.810 pregnant singleton women were evaluated at the time of a 75 g OGTT between 24^0^ and 28^6^ weeks for the universal screening of gestational diabetes (GDM). The 75 g OGTT was considered flat when the difference between peak and fasting glucose concentrations was ≤30 mg/dL. A total of 953 (10.8%) women were diagnosed as having GDM, while 7.857 (89.2%) had normal glucose tolerance (NGT); 2791 women with normal glucose tolerance (35.5%) had a FLAT curve and 5066 (64.5%) had a concentration difference > 30 mg/dL (NGT). In all groups, we evaluated maternal characteristics and perinatal outcome. **Results**: Women with a FLAT curve were younger, taller, thinner, and their pre-pregnancy body mass index was lower than the other groups (all *p* < 0.001). The rate of obesity was also lower (*p* < 0.01). The vaginal delivery rate was higher than in NGT (80.4% vs. 77.8%; *p* < 0.01) and women with GDM (73.0%; *p* < 0.001) and that of primary cesarean lower than in NGT (11.9% vs. 14.8%; *p* < 0.001) and women with GDM (18.2%; *p* < 0.001). Between women with a FLAT and NGT OGTT curve, there was no significant difference for birthweight < 10th percentile (6.9% vs. 6.2%; *p* = 0.2), though the proportion of birthweight > 90th was lower (8% vs. 10%; *p* < 0.01). **Conclusions**: A 75 g flat OGTT as defined does not represent an abnormal maternal phenotype nor portend an adverse perinatal outcome.

## 1. Introduction

Virtually all obstetrical societies and academic institutions providing antenatal care for pregnant women recommend testing for glucose intolerance in pregnancy, using an oral glucose tolerance test (OGTT) [1,2,3,4]. Based on WHO criteria [4], a 2 h 75 g glucose OGTT is recommended at 24^0^–28^6^ weeks gestation. In women with normal glucose tolerance (NGT), the 1 h glucose value < 180 mg/dL is expected, followed by a decrease to <153 mg/dL, by the end of the second hour. However, it is not uncommon to observe that at 1 h there may be little or no increase in plasma glucose concentration, often defined as a flat glucose curve [5,6,7,8,9,10,11].

The clinical significance of the flat curve is not univocal: some consider it representative of the presence of malabsorption [6,12]; others suggest elevated levels of insulin [5,7,9,13]; and some as a variant of the normal response [5]. Although flat glucose curves have been described in various clinical situations, such as hypothyroidism and idiopathic steatorrhoea [5,10,13,14], there is no consensus on either the criteria or the significance of the observation of a flat OGTT curve.

In pregnancy, maternal low concentrations of glucose during the OGTT have been associated with fetal growth restriction or small for gestational age (SGA) neonates in many studies [15,16,17,18,19,20,21,22,23,24,25,26,27,28,29]. However, there has been no consensus of how to define a flat OGTT, and the cut-off values for the definition of a flat OGTT differed among the various studies.

Therefore, the aim of this study was to compare the obstetric characteristics and the clinical outcomes of women with a flat OGTT compared to (1) women with a positive OGTT, i.e., gestational diabetes (GDM), and (2) women with a normal OGTT but not meeting the criteria of a flat OGTT curve. To define an OGTT curve as flat, we modified the criteria proposed by Thaysen and Norgaard [12], i.e., an OGTT curve that, after a 100 g glucose challenge, shows a rise of ≤40 mg/dL after 1 h compared to the fasting glucose. However, since we included only women who ingested 75 g of glucose, we utilized the proportionally reduced value of ≤30 mg/dL to define women with a flat OGTT.

## 2. Materials and Methods

### 2.1. Study Population

We retrospectively analyzed the obstetric outcome of term, singleton pregnancies who delivered at the Unit of Obstetrics and Gynecology of the S. Paolo Hospital Medical School, Milan, Italy, since 2013, when we introduced the 75 g OGTT instead of the two-step Carpenter and Coustan approach [30] for the universal screening of GDM. Only women with livebirths without chromosomal abnormalities and/or major malformations were included. A total of 8810 women were identified with a complete 75 g OGTT performed between 24^0^ and 28^6^ weeks of gestation. The OGTT was performed after an overnight fast of at least 8 h. GDM was diagnosed in 953 (10.8%) women. The diagnosis was made using the WHO criteria [4] when any one of the following plasma glucose values were ≥92 mg/dL at fasting; ≥180 mg/dL after one hour; ≥153 mg/dL after two hours (GDM group). Conversely, in 7857 (89.2%), the OGTT was considered normal when all the values were less than the WHO criteria (NGT group). In women with NGT, we then examined the peak glucose value at one hour and compared this with the fasting or basal glucose concentrations. Women considered to have a flat OGTT were those defined as those with a concentration difference of ≤30 mg/dL between the fasting and 1 h glucose (2791 women, 35.5%; FLAT group). The NGT group was thus composed of women whose concentration difference was >30 mg/dL (5066 women, 64.5%; NGT group).

In each group, we evaluated woman’s age, parity, ethnicity, height, pre-pregnancy weight, body mass index (kg/m^2^), gestational weight gain, smoking, pregnancy conceived with assisted reproduction (ART), presence of treated thyroid disease or hypertension, malabsorption, mode of delivery, newborn weight and length, birthweight percentiles according to the Intergrowth standards [31], ponderal index (grams neonatal weight/neonatal length^3^ × 100), Apgar scores, umbilical artery pH, and base deficit at delivery.

The study was exempt from Institutional Review Board approval because obstetric and neonatal outcomes were collected as part of the clinical management. The privacy of all patients was maintained.

### 2.2. Statistics

Categorical variables were described as frequency (N) and percentages (%) and compared using chi-square test or Fisher’s Exact Tests, as appropriate. Continuous variables were described as mean and standard deviation (SD) and for normally distributed data analyzed using Student’s *t*-test. In addition, a robust multivariate logistic model was fitted to the data considering vaginal as compared with cesarean deliveries as the outcome variable; the predictors were maternal body mass index, maternal age, birthweight, and flat OGTT curve expressed as dichotomous variable (flat vs. non-flat OGTT). These measures were included in the multivariate logistic model because of the increased risk of cesarean deliveries associated with higher maternal BMI, age, and birthweight. We wanted to adjust for these well-recognized predictors to determine the additional risk of cesarean delivery with a flat OGTT. The goodness of fit of this model was assessed by Hosmer–Lemeshow’s test. The significance threshold was set at 0.05 for all calculations. The analyses were performed with STATA^®^18 for MacOS (STATACorp., Inc., College Station, TX, USA).

## 3. Results

### Participant Characteristics

The OGTT was performed at 25.8 ± 1.3 weeks with no significant differences among the GDM, NGT, and FLAT groups. Table 1 presents the fasting glucose concentration, one and two hours after the oral glucose load, the difference between the peak and the fasting value, and the OGTT area under the curve (AUC) in the three groups, showing that all parameters were significantly lower in FLAT when compared to NGT and GDM women. Table 1 also presents the clinical characteristics of the women in the three groups. Women in the FLAT group were younger, taller, and had a lower BMI than women with NGT and GDM. The rate of obesity was significantly lower in FLAT than in NGT and GDM groups, and that of underweight highest in the FLAT group.

The proportion of women who conceived by assisted reproduction was also significantly lower in the FLAT group. Obstetric outcomes are presented in Table 2.

The rate of pre-gestational and gestational hypothyroidism was significantly lower in the FLAT group when compared with women with GDM; similarly, the rate of hypertension was significantly lower in women in the FLAT group. We did not find any significant differences in medical conditions (i.e., inflammatory bowel disease) potentially relating to malabsorption in the three groups (0.9%, 1.3%, 1.1%, respectively, in NGT, FLAT, and GDM groups). The rate of vaginal delivery was significantly higher and that of primary cesarean significantly lower in women in the FLAT group, compared with the other two groups. Newborn weight in the FLAT group was significantly lower than the newborn weight of women with NGT. The proportion of newborns > 90° percentile was also lower in women in the FLAT group; however, there was no significant difference noted for newborns < 10° percentile. Neonatal length in the NGT and FLAT groups was longer than in neonates of women with GDM. There were no significant differences in newborn ponderal index. At the time of delivery, Apgar scores at 5′, umbilical arterial pH, and base deficit were also similar in the three groups. Overall, only 24 neonates had a 5′ Apgar score < 7 and 42 pH values < 7.0, with no significant difference among groups. Table 3 presents the results of the multivariate logistic analysis showing that the presence of a flat OGTT curve, as defined, was an independent predictor of both vaginal delivery and primary cesarean.

**Figure 1:**
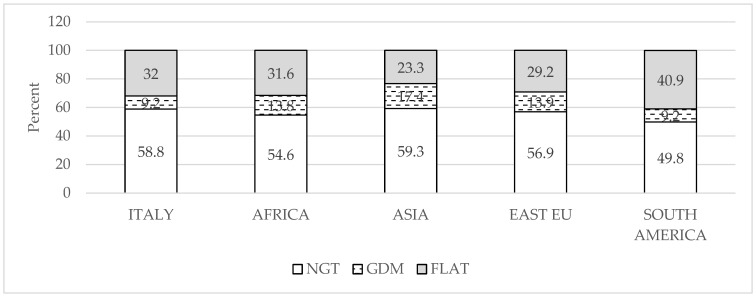
Percent of women with normal glucose tolerance (NGT, open bar), gestational diabetes (GDM, dashed bar), and flat OGTT curve (FLAT, gray bar) according to self-reported ethnicity.

Since in our unit we have a health center for immigrant women who represent 35.4% of the total population, we analyzed the distribution of the different OGTT results according to the different ethnic groups. Figure 1 shows that women from South/Central America had the highest rate of a flat OGTT curve and the lowest rate of GDM compared to women from Italy, Africa, Asia, and East Europe.

## 4. Discussion

The results of our study show that women with a flat OGTT have a healthy demographic profile and less adverse pregnancy outcomes, such as a lower rate of newborns large for gestational age (>90° percentile) compared with NGT and no significant increase in newborns small for gestational age (<10° percentile) as compared with both NGT and GDM. A flat OGTT may represent a carbohydrate metabolic profile of decreased insulin resistance and/or robust pancreatic beta cell function as evidenced by a significant decreased glucose AUC during the OGTT compared with NGT and GDM. Supporting this hypothesis are the results of the recent study by Tarashandegan et al. [32], reporting that women with a flat 100 g OGTT during pregnancy [fasting value below 95 mg/dL and the other three below 100 mg/dL] exhibit a significantly low risk of developing type 2 diabetes up to 5 years following pregnancy compared with those with NGT and glucose values > 95 mg/dL fasting and 100 mg/dL post glucose challenge.

The concept of a flat OGTT has been a recurrent theme in the literature since at least 1929, when Thaysen and Norgaard [12] observed that individuals with idiopathic steatorrhea had what he referred to as “low blood sugar curves”, defined as a rise of 40 mg/dL or less after a 100 g glucose load. Thaysen later [14] hypothesized that “low blood sugar curves” were related to pathologies such as celiac disease or sprue. Using the same definition as Thaysen, Lepore [6] reported that, in individuals affected by sprue, the flat curve was correlated with the intake of a diet rich in carbohydrates. Thirty years later, Das Gupta, observing a flat curve in 20% of people without malabsorption or hypothyroidism undergoing an OGTT for various reasons, hypothesized that it was a variant of the normal OGTT curve [5].

Over time the term “flat curve” has often conflated with that of “reactive hypoglycemia” based on a series of studies showing a correlation of reactive hypoglycemia with SGA or growth-restricted babies. However, there is a wide variability in the definition of adverse neonatal outcomes from birthweights ≤ 10th percentile [17] to growth restrictions; either with low or adequate birthweight [22], or from birthweight < 2500 g [18]. Others have used an increased number of Neonatal Intensive Care Unit admissions [21] as an adverse perinatal outcome measure. Additionally, many of the studies used various glucose loads during the OGTT, creating further confusion in the definition of a flat curve: from glycemia ≤ 50 mg/dL [28], or <100 mg/dL after a 100 g load [7], to glycemia < 88 mg/dL [21] or <108 [23] after a 50 g glucose challenge test, to glycemia < 63 mg/dL after a 75 g load [25,29]. However, we believe that defining a flat OGTT curve as hypoglycemic may be misleading. In 43 pregnant women at risk of intrauterine growth restriction, Langer et al. [15] found that in maternal hypoglycemia, as expressed by the glucose index (i.e., the sum of the first, second, third and fourth hour, post 100 g glucose load values, minus fasting plasma glucose), less than 105 mg/dL was significantly associated with SGA infants in absence of hypertension. The Langer group also measured insulin and, interestingly, found lower levels compared with NGT and appropriately grown babies. A potential explanation was postulated by Cryer et al. [33]. He reported, in normal non-pregnant adults, that when glycemia falls below the threshold value of 67 mg/dL, necessary for the maintenance of brain metabolism, the prevention and correction of hypoglycemia results in a reduction in insulin and activation of the glucose response systems, which may possibly explain the significance of the previous observations. However, when we calculated the glucose index in our population, we found that 94% of women with a glucose index < 105 mg/dL had a flat OGTT curve that, in contrast, characterizes a population with a more favorable outcome, when compared to women with NGT and GDM. These differences may relate to a 75 g and 100 g glucose load and of non-insulin-mediated glucose transport from mother to the feto-placental unit.

Recently, studies from Israel have examined the relationship between a flat OGTT and the perinatal outcomes in pregnant women [7,32,34]. The flat OGTT curve was defined as a fasting value below 95 mg/dL and the other three below 100 mg/dL, The results of these studies are very similar with 4.7%, 6.4% and 6.8% of women, respectively, having a flat OGTT curve as described; in addition, similarly to the results of the present study, women with a flat OGTT curve were younger and leaner and had better perinatal outcomes than women with a normal glucose tolerance test curve [7,32,34].

Interestingly, in our study, using their same definition of flat OGTT curve, 11.4% of women have an OGTT where none of the values exceeds 100 mg/dL [68% of the FLAT group]. In our report, however, only 7% of women with a flat OGTT curve have a BMI > 30 kg/m^2^, while in Lopian’s study they found 24% [34], and in Naeh’s study the mean BMI is 29.4 ± 5.9 kg/m^2^ [7]. These differences in BMI might explain the difference in the results among the studies. Furthermore, according to the WHO criteria [4] in which the cut-off of the basal glucose value is ≤92 mg/dL, the presence of fasting glucose < 95 mg/dL, as in the previous studies [7,32,34], might have included in the flat OGTT curve group of women that with the 75 g OGTT would have been considered as GDM. Hence the importance of knowing which glucose load is used in the definition of GDM.

Another recent study by Navon et al. [8] defined a 100 g OGTT curve where the area under the curve was ≤10th percentile of the study population as flat. In their study, 10% of women had a flat OGTT curve. Similarly to our study, women in the FLAT group were younger and had a lower BMI than women in the NGT group. In contrast, there was a higher proportion of SGA newborns, although evaluated using a different reference standard than ours [31]. Nevertheless, Navon also reports no differences in obstetric outcomes [8]. Last, Szoke et al. [11] also addressed the issue of the flat OGTT curve in pregnancy, defined with a complex calculation, as the increase in glucose peak < 6% compared to fasting glucose. In their study, 5.8% of women had a flat OGTT curve. Since insulin and c-peptide concentrations were also measured, they hypothesized that a flat OGTT curve is due to the presence of a hypersensitivity to insulin, with normal beta cell function in these women. However, the obstetric outcomes were not included in their study.

Pregnancy is a unique condition regarding carbohydrate metabolism. Although pregnancy is characterized by lower fasting glucose with advancing gestation and hyperinsulinemia [35], there is a 30% increase in hepatic glucose production during pregnancy, representing hepatic insulin resistance [36]. The metabolic goal of the increase in hepatic glucose production is to provide glucose as the main metabolic energy substrate for both the fetus and placenta [37]. The flat OGTT could mean higher insulin sensitivity compared to women with normal glucose tolerance, due to lower BMI; unfortunately, since this is a retrospective study and the concentration of insulin was not measured, its mechanism in flat OGTTs was not clarified.

In summary, the current study addresses a clinical situation that is not uncommon in pregnancy: the lack of a glycemic peak after an OGTT. Unfortunately, there is no consensus on the definition and interpretation of the phenomenon. Our study shows that a definition easy to calculate and remember, i.e., a difference between peak and fasting glucose ≤ 30 mg/dL, is associated with improvement in the perinatal outcomes both when compared to NGT pregnancies with a difference > 30 mg/dL in fasting and 1 h glucose after a 75 g OGTT, and to pregnancies complicated by GDM. Because this is a retrospective study, there are questions that remain open in women with a flat OGTT curve. What would the curve be like if repeated at a different gestational age? What are the factors that determine the response, including, but not limited to, insulin concentration, diet, and physical activity? Only a prospective study, in which other factors implicated in glucose metabolism in pregnancy are evaluated, will be able to answer these questions.

### Strengths and Limitations

The primary limitation of this study is that it is retrospective. Another theoretical concern is that the insulin concentrations are not routinely measured to estimate insulin sensitivity and response. We speculate that women with a flat OGTT curve have higher insulin sensitivity, a robust pancreatic beta cell function and healthy eating index (HEI), and higher levels of physical activity. Unfortunately, these are not routine measures in clinical practice but would be helpful in future studies to understand potential mechanisms in developing an optimal metabolic profile to decrease adverse pregnancy outcomes. In contrast, a major strength is the large number of women analyzed, with ethnic diversity, followed in the same institution with the same protocols. Furthermore, for the definition of the flat OGTT curve we propose a value that is easily measured, i.e., the difference between peak and fasting glucose ≤ 30 mg/dL, and easy to remember.

## 5. Conclusions

The results of our study show that the lack of a glucose peak at the time of the oral glucose tolerance test during pregnancy; that is, a flat OGTT curve, as defined, i.e., a difference between peak and fasting glucose ≤ 30 mg/dL, does not represent an abnormal maternal phenotype nor an adverse obstetric and neonatal outcome. Based on our results, it appears that no additional clinical efforts need be initiated in individuals with a flat 75 g OGTT curve of ≤30 mg/dL, as it does not portend adverse perinatal outcomes.

## Figures and Tables

**Table 1:** The clinical characteristics of the women in the three groups. Data are mean ± SD or number and percentage, as appropriate.

	NGT N = 5066	GDM N = 953	FLAT N = 2791	NGT vs. GDM	NGT vs. FLAT	FLAT vs. GDM
G T0, mg/dL	77.8 ± 6.6	88.4 ± 10.4	77.5 ± 6.1	0.001	0.04	0.001
G T60, mg/dL	132.7 ± 18.3	166.0 ± 32.7	92.0 ± 13.4	0.001	0.001	0.001
G T120, mg/dL	107.4 ± 19.6	143.7 ± 40.7	90.4 ± 17.0	0.001	0.001	0.001
G T60–T0, mg/dL	55.0 ± 17.1	77.5 ± 35.8	14.5 ± 11.9	0.001	0.001	0.001
G AUC mg/dL	317.9 ± 35.1	398.1 ± 56.6	259.8 ± 27.9	0.001	0.001	0.001
Age, years	32.1 ± 5.5	33.0 ± 5.6	30.9 ± 5.8	0.001	0.001	0.001
Age ≥ 35 years (%)	1807 (35.7)	397 (41.7)	795 (28.5)	0.001	0.001	0.001
Height, cm	163.1 ± 6.6	162.3 ± 6.5	163.7 ± 6.5	0.002	0.001	0.001
Weight, kg	62.8 ± 12.6	65.6 ± 13.3	61.5 ± 12.1	0.001	0.001	0.001
GWG, kg	12.4 ± 4.6	10.3 ± 5.1	12.7 ± 4.5	0.001	0.02	0.001
BMI, kg/m^2^	23.6 ± 4.5	24.9 ± 5.0	23.0 ± 4.3	0.001	0.001	0.001
Underweight, (%)	363 (7.4)	51 (5.6)	255 (9.5)	0.05	0.002	0.001
Obese (%)	460 (9.3)	140 (15.3)	202 (7.5)	0.001	0.01	0.001
Gravida 1, (%)	1818 (35.9)	308 (32.3)	980 (35.1)	0.04	0.5	0.1
Immigrants (%) non Italian	1725 (34.1)	430 (45.1)	966 (34.6)	0.001	0.6	0.001
Tobacco use (%)	393 (11.2)	65 (10.8)	216 (10.9)	0.8	0.8	1.0
ART (%)	220 (4.3)	46 (4.8)	77 (2.8)	0.5	0.001	0.003

NGT = normal glucose tolerance; GDM = gestational diabetes; BMI = body mass index calculated on pre-pregnancy weight available in 4933 NGT; 918 GDM; 2696 FLAT; GWG = gestational weight gain available in 4393 NGT; 777 GDM; 2448 FLAT; smoking available for 3522 NGT; 1990 FLAT; 605 GDM; ART = assisted reproduction technique; G = glucose; AUC = area under the curve.

**Table 2:** The obstetric outcome of the women in the three groups. Data are mean ± SD or number and percentage, as appropriate.

	NGT N = 5066	GDM N = 953	FLAT N = 2791	NGT vs. GDM	NGT vs. FLAT	FLAT vs. GDM
GA at delivery, weeks	39.5 ± 1.1	39.0 ± 0.9	39.6 ± 1.1	0.001	0.04	0.001
Hypothyroidism (%)	510 (10.1)	111 (11.6)	254 (9.1)	0.3	0.2	0.03
Hypertension (%)	239 (4.7)	57 (6.0)	101 (3.6)	0.1	0.02	0.003
Induction of labor (%)	1401 (27.7)	402 (42.2)	751 (26.9)	0.001	0.5	0.001
Vaginal delivery (%)	3939 (77.8)	696 (73)	2244 (80.4)	0.002	0.01	0.001
Primary cesarean (%)	654 (14.8)	146 (18.2)	290 (11.9)	0.01	0.001	0.001
Blood loss at VD, mL	373 ± 378	351 ± 291	354 ± 322	0.1	0.02	0.9
>500 mL (%)	19.4	16.3	17.1	0.06	0.03	0.6
Newborn weight, g	3327 ± 419	3269 ± 411	3298 ± 410	0.001	0.003	0.06
>90° centile (%)	506 (10)	96 (10.1)	224 (8.0)	0.1	0.01	0.06
<10° centile (%)	312 (6.2)	59 (6.2)	193 (6.9)	0.9	0.2	0.5
Newborn length, cm	50.3 ± 1.9	50.0 ± 1.8	50.2 ± 1.9	0.001	0.09	0.005
Newborn Ponderal Index, g/l^3^	2.6 ± 0.3	2.6 ± 0.2	2.6 ± 0.3	0.1	0.08	0.7
Apgar at 5′	9.9 ± 0.4	9.9 ± 0.4	9.9 ± 0.4	0.9	0.2	0.5
Umb artery pH	7.264 ± 0.09	7.267 ± 0.09	7.266 ± 0.09	0.4	0.4	0.8
Umb artery BD, mM	−4.9 ± 3.5	−4.6 ± 3.4	−4.8 ± 3.4	0.03	0.4	0.1

VD = vaginal delivery; newborn length and ponderal index available in 2850 NGT; 1520 FLAT; 568 GDM; umbilical artery pH and BD available in 4568 NGT; 2489 FLAT; 862 GDM.

**Table 3:** The results of the multivariate logistic analysis.

Variable	OR [95% CI]	*p*
Predictors of vaginal delivery		
Flat curve	1.13 [1.01–1.26]	0.034
Maternal age, years	0.97 [0.96–0.98]	0.001
Maternal body mass index, kg/m^2^	0.94 [0.93–0.95]	0.001
Birthweight, kg	1.0005 [1.0003–1.0006]	0.001
Predictors of primary cesarean		
Flat OGTT	0.83 [0.72–0.96]	0.016
Maternal age, years	1.04 [1.03–1.05]	0.001
Maternal body mass index, kg/m^2^	1.02 [1.01–1.03]	0.001
Birthweight, kg	0.9995 [0.9994–0.9997]	0.001

## Data Availability

The data presented in this study are available on request from the corresponding author. Data are unavailable due to privacy restrictions.

## Abbreviations

The following abbreviations are used in this manuscript:

ART	Assisted Reproduction Technique
AUC	Area Under the Curve
BMI	Body Mass Index
GA	Gestational Age
GDM	Gestational Diabetes Mellitus
GWG	Gestational Weight Gain
HEI	Healthy Eating Index
NGT	Normal Glucose Tolerance
OGTT	Oral Glucose Tolerance Test
SGA	Small for Gestational Age
VD	Vaginal Delivery
WHO	World Health Organization
